# Effects of Ascorbic Acid on Osteopontin Expression and Axonal Myelination in the Developing Cerebellum of Lead-Exposed Rat Pups

**DOI:** 10.3390/ijerph16060983

**Published:** 2019-03-19

**Authors:** Sung Min Nam, Jin Seok Seo, Sang-Soep Nahm, Byung-Joon Chang

**Affiliations:** Department of Anatomy, College of Veterinary Medicine, Konkuk University, Seoul 05030, Korea; skavet@konkuk.ac.kr (S.M.N.); phoenix_1st@naver.com (J.S.S.); ssnahm@konkuk.ac.kr (S.-S.N.)

**Keywords:** lead (Pb) toxicity, ascorbic acid, cerebellum, osteopontin, oligodendrocyte, locomotive test

## Abstract

Osteopontin (OPN) is a multi-functional protein that binds to integrin and calcium-binding phosphoprotein. OPN is required for normal neuronal development and its axonal myelination. We studied the combined effect of lead (Pb) and ascorbic acid treatment on OPN expression in the developing cerebellum. We randomly divided pregnant female rats into three groups: control, Pb (lead acetate, 0.3%, drinking water), and Pb plus ascorbic acid (PA; ascorbic acid, 100 mg/kg, oral intubation) groups. The blood level of Pb was significantly increased, while ascorbic acid reduced Pb levels in the dams and pups. At postnatal day (PND) 21, results from Nissl staining and OPN immunohistochemistry demonstrated that OPN was detected in the Purkinje cell layer in the cerebellum. Ascorbic acid treatment mitigated Pb exposure-induced reduction in the number of intact Purkinje cells and OPN immunoreactive Purkinje cells in the cerebellum of pups. In addition, Pb-induced reduction in the number of oligodendrocytes and myelin-associated glycoprotein is associated with the malformation of the myelin sheath. Ascorbic acid provided protection from Pb-induced impairments. Pb-induced structural deficits in the cerebellum resulted in functional deterioration observed during locomotive tests (bar holding test and wire mesh ascending test), while ascorbic acid ameliorated these harmful effects. Present results suggest that the change of OPN is associated with myelination in the developing cerebellum. The results also demonstrated that exposure to Pb is harmful, while ascorbic acid treatment is beneficial.

## 1. Introduction

Osteopontin (OPN) is known as a glycosylated phosphoprotein with calcium- and integrin-binding capabilities [1]. Alternative splicing generates various forms of OPN; these are associated with numerous functions including inflammatory response [1,2], tissue mineralization [1,3], cancer and metastatic proficiency [4], tissue repair [5], and development [6]. OPN is widely expressed in the bone, kidney, breast, thymus, testis, immune system, spinal cord, and brain [1,7,8,9,10].

Until recently, research on OPN has focused on its pathophysiological role. In particular, OPN is considered as an inflammatory mediator in various neurodegenerative diseases such as multiple sclerosis [11,12], Alzheimer’s disease [13], and Parkinson’s disease [14]. However, importantly, Jiang et al. [15] recently described the functional role of OPN in brain development. Specifically, they focused on the fact that OPN is transferred from the mother to the offspring through the milk produced by the mother, and that OPN is produced in the mammary glands and the brain [15]. When ingested, iodine-labeled OPN in milk can reach the offspring’s brain resulting in an increase of OPN levels in the brain of the offspring [15]. The early postnatal period is critical for the rodent brain’s development and maturation, and we previously reported that lead (Pb) exposure impairs the normal development of the hippocampus and cerebellum [16,17]. Pb can also be transferred from dam to offspring via the placenta and milk produced by the dam [18]. Pb exposure is detrimental to the myelination of fibers and Purkinje cell development in the cerebellum. OPN is one of the possible mediators of myelination and neuronal differentiation in the brain [15,19,20]. Therefore, we hypothesize that there may be an association between Pb-induced hypomyelination, a reduction in neuron numbers, and OPN expression. 

Ascorbic acid effectively attenuates the abnormal development of the brain induced by Pb poisoning [16,17,20]. Ascorbic acid is also functionally important during developmental processes including neuronal differentiation and synaptic maturation [21]. To observe the combined effects of Pb and ascorbic acid treatment on cerebellar development in the offspring from exposed individuals, we investigated neuronal development, oligodendrocytes, myelination, and OPN expression in the cerebellum.

## 2. Materials and Methods 

### 2.1. Experimental Design and Animals

Sprague Dawley (SD) rats purchased from Narabiotec Co., Ltd (Seoul, Republic of Korea) were used in our present study. The experimental protocol was approved by the Institutional Animal Care and Use Committee of the Konkuk University (approval number, KU18133). Female (*n* = 9) and male (*n* = 3) rats (8 weeks old) were housed under a constant condition, with a temperature of 22 to 24°C, humidity (60%), and illumination (12:12h light/dark cycle). The animals were acclimated to a conventional state at the animal facility in the College of Veterinary Medicine and were then used for the experiments. Female rats were confirmed to be pregnant when vaginal plugs were present or when sperm was detected on vaginal smears. According to the previous our study [17], day 0 was designated and pregnant female rats were singly caged until the end of the experiments. Animal handling and caring followed the Guide for the Care and Use of Laboratory Animals, which was issued by the Institute of Laboratory Animal Resources, National Institutes of Health, USA, 1996. 

### 2.2. Chemical Treatment

Female SD rats were randomly divided into three groups: the control group (*n* = 3), Pb group (*n* = 3), and Pb plus ascorbic acid (PA) group (*n* = 3). The design of the experiment and the administered doses of Pb and ascorbic acid were prepared as reported in previous studies [16,17,22]. Lead acetate (Pb(C_2_H_3_O_2_)_2_, 0.3% in distilled water; (Sigma-Aldrich, St. Louis, MO, USA)) was prepared with glacial acetic acid (0.05%; Junsei Chemical Co., Tokyo, Japan) to prevent Pb precipitation. Ascorbic acid (100 mg/kg; Sigma-Aldrich), which was freshly prepared in saline, was orally administered. To account for the stress during oral intubation, female rats in the control and Pb groups only received the same volume of saline. From one week prior to the mating day, Pb and ascorbic acid treatments started and were carried out during gestation and delivery of pups until the end of the experiment. The body weights of offspring were measured and averaged every week. To preclude any effect concerning litter size during the experiment, eight rat pups per dam (total 24 pups per group) were selected for further analysis and the remaining pups were sacrificed. Whenever possible, only male pups were used for the experiment and female pups were used only to maintain equivalent litter sizes. The experimental procedures were carefully conducted to minimize suffering and the number of animals used.

### 2.3. Locomotor Coordination Assay (Bar Holding Test and Wire Mesh Ascending Test) in Offspring

On postnatal day (PND) 19, 12 offspring per group were assessed for motor function using a modified protocol described in a study by Perez-Polo et al. [23]. During the bar holding test, pups were allowed to grasp a stainless-steel bar (0.7 cm diameter × 35 cm length) that was suspended 30 cm over a soft surface. The duration of time spent with forelimbs grasping the bar was measured within 60-s deadline. During the wire mesh ascending test, a 0.7-cm thick stainless-steel mesh (45 cm length× 30 cm width) was placed at an angle of 26° in a water bath containing water at 25 °C such that the wire mesh was 30 cm above the water surface. The offspring was placed with its quarter hind and tail dipped in the water. After three separate training trials, the achievement time, which is the time taken for the pup to reach the top of the mesh, was recorded over a 30-s period.

### 2.4. Analysis of Pb Level in Blood Using Atomic Absorption Spectrometry and Measurement of Cerebellar Weight

On PND21, three dams per group, and 12 offspring per group, were anesthetized by the intraperitoneal injection of urethane (1.5 g/kg; Sigma-Aldrich). Blood, for analysis of Pb levels, was drawn from left ventricle separately from dams and pups by cardiac puncture and analyzed using an atomic absorption spectrophotometer (Perkin Elmer Zeeman 5100; Norwalk, CT, USA) and an HGA-600 graphite furnace with Zeeman background correction. The absorption wavelength was 283.3 nm and the r2 of the calibration curve exceeded 0.995. The weights of the cerebellum of offspring were measured on PND21.

### 2.5. Tissue Processing and Histological Analysis

For histological evaluation, the procedures for Nissl staining and immunohistochemistry were used, as described in our previous studies [22,24]. Briefly, the remaining 12 offspring (*n* = 12 per group) were anesthetized by urethane on PND21 and perfused transcardially with heparinized phosphate-buffered saline (PBS; 0.1 M, pH 7.4), followed by fixative (4% paraformaldehyde in 0.1 M phosphate buffer, pH 7.4). The cerebella were dissected out and post-fixed in the same fixative overnight at 4 °C. Cerebellar tissues were embedded in paraffin. Mid-sagittal sections (5-μm) of the vermis were selected and a total of 36 paraffin sections per group (three sections per offspring) were used. Deparaffinized sections were placed in citrate buffer (pH 6.0) for antigen retrieval. The sections were then quenched with 0.3% hydrogen peroxide (H_2_O_2_) and incubated with 10% normal horse serum for blocking. Afterward, the sections were kept overnight at 4 °C in goat anti-osteopontin (OPN, 1:500; R&D systems, Minneapolis, MN, USA), goat anti-oligodendrocyte transcription factor 2 (Olig2, 1:500; R&D systems), or mouse anti-myelin associated glycoprotein (MAG, 1:1000; Millipore, Billerica, MA, USA). Subsequently, sections were exposed to biotinylated immunoglobulin G (1:200; Vector, Burlingame, CA, USA) and streptavidin peroxidase complex (1:200; Vector). Reactive sites were visualized with 3,3′-diaminobenzidine tetrachloride (Sigma-Aldrich) in 0.1 M Tris-HCl buffer (pH 7.2). Following dehydration, sections were mounted in a toluene-based mounting medium (Richard-Allan Scientific, Thermo Scientific, Waltham, MA, USA). 

Histopathological analyses were performed by investigators blinded to treatments. The numbers of Olig2-positive cells in all groups were counted using an image analysis system equipped with a computer-based CCD camera (Optimas 6.5 software, CyberMetrics, Scottsdale, AZ, USA). The quantification method of Purkinje cells employed in the present study was modified from that used in a previous study [22]. The Purkinje cells were counted in micrographs obtained at 100× magnification per 5000 μm length of Purkinje cell layer using the arbitrary line probe of the DP2-BSW software (Olympus, Tokyo, Japan). The observations were carried out in the 2nd, 5th, and 8th lobules of sagittal sections of the cerebellar vermis [25]. Ten lobules of cerebellum disappeared from the midline sagittal sections to lateral sagittal sections and we excluded the sections when the number of cerebellar lobules is reduced from ten. Therefore, the number of Purkinje cells was demonstrated and compared as a relative number of the control group.

Analysis of a region of interest in the cerebellum was performed by calibrating the image into an array of 512 × 512 pixels corresponding to a tissue area of 140 × 140 μm (40 × primary magnification). Each pixel resolution was 256 gray levels. The intensity of myelin-associated glycoprotein (MAG) immunoreactivity was evaluated by means of a relative optical density (ROD), which was obtained after the transformation of the mean gray level using the following formula: ROD = log (256/mean gray level). Using NIH Image 1.59 software, ROD of the background was determined in unlabeled portions, and the value was subtracted for correction, yielding high ROD values in the presence of preserved structures and low values after a structural loss. A ratio of the ROD was calibrated as a percentage.

### 2.6. Statistical Analysis

Data for each group are expressed as means ± standard errors of the mean, and the significance of the differences was determined using one-way analysis of variance followed by Bonferroni’s post hoc test for multiple comparisons. Data were analyzed by GraphPad Prism 5.01 software (GraphPad Software, Inc., La Jolla, CA, USA). The significance in differences were set at *P*-values <0.05.

## 3. Results

### 3.1. Body Weight, Cerebellar Weight, and Blood Pb Levels

**Figure 1:**
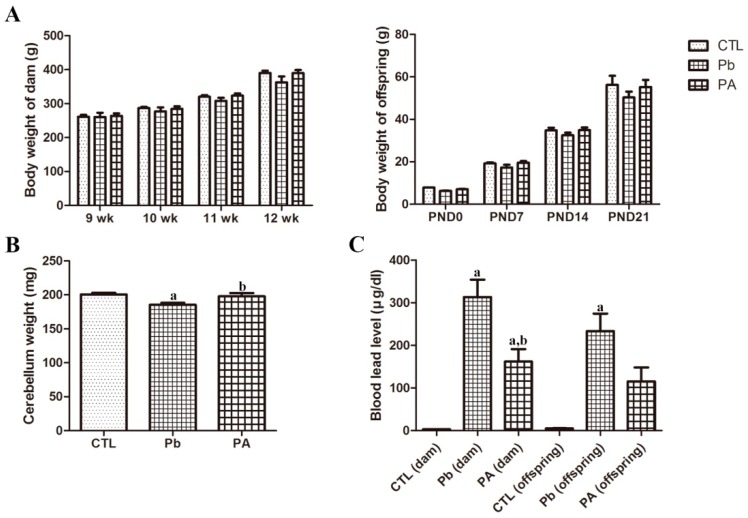
Body weights of dams (*n* = 3 per group) during gestation and offspring (*n* = 12 per group) on postnatal days (PND) 0, 7, 14, and 21 in control (CTL), lead (Pb), and Pb+ ascorbic acid (PA) groups (**A**). Cerebellar weights of offspring on PND21 (**B**). Blood Pb levels in the dams and offspring on day 21 after delivery (**C**) (^a^ *p* < 0.05, indicating a significant difference compared with the control group, ^b^ *p* < 0.05, indicating a significant difference compared with the Pb group). The bars indicate means ± standard errors of mean.

At the end of the experiment, the body weights of dams and offspring reduced by long-term exposure to Pb with no statistical significance (*p* > 0.05). However, ascorbic acid co-treatment with Pb attenuated the Pb-induced decrease in body weight. Pb exposure during pregnancy and lactation also prominently reduced the weight of cerebellum (*p* < 0.01), while ascorbic acid co-treatment ameliorated Pb-induced weight changes at PND21 (*p* < 0.05). Atomic absorption spectrometry confirmed that blood Pb levels in dams and offspring were increased by long-term Pb exposure(dam 313.513 μg/dL, *p* < 0.01; offspring 233.25 μg/dL, *p* < 0.01),while ascorbic acid co-administration reduces the Pb levels (dam 161.929 μg/dL, *p* < 0.05; offspring 115.084 μg/dL, *p* > 0.05)(Figure 1).

### 3.2. Effects of Pb Exposure and Ascorbic Acid Treatment on Cerebellar Development

**Figure 2:**
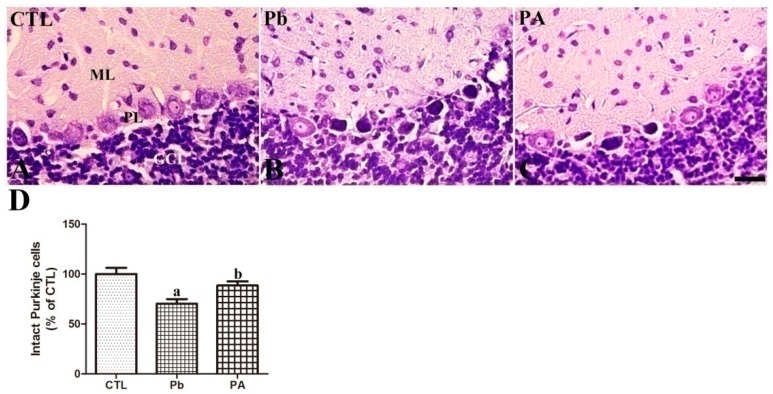
Nissl staining of cerebellum (**A**–**C**) of offspring from CTL, Pb, and PA groups. GCL, granule cell layer; ML, molecular layer; PL, Purkinje cell layer. Bar = 25 μm. (**D**) The numbers of intact Purkinje cells in the cerebellum are expressed as percentages of the value in the CTL group. The number of intact Purkinje cells in three sections per offspring in non-overlapping fields, 12 rats were included in each group (*n* = 12 offspring per group; ^a^ *p* < 0.05, indicating a significant difference compared with the control group, ^b^ *p* < 0.05, indicating a significant difference compared with the Pb group). The bars indicate the means ± standard errors of the mean.

Nissl staining was conducted to evaluate the effect of Pb exposure and ascorbic acid treatment on the developing cerebellum. On PND21, three layers of the cerebellar cortex were examined in all groups. In the cerebellum at PND21, long-term Pb exposure caused a significant reduction in the number of intact Purkinje cells (rim of cytoplasm surrounding nucleus with cytoplasmic extension to dendrites) to 70.33% of that in the control group (*p* < 0.01), while increasing the number of degenerating Purkinje cells (pyknotic or vacuolated changes in Purkinje cell layer). Among degenerating changes, pyknotic cells were observed in the Pb group. Ascorbic acid co-treatment ameliorated Pb-induced reduction in the number of intact Purkinje cells to 88.52% of that in the control group (*p* < 0.05). The mean number of intact Purkinje cells in the PA group was less than that observed in the control group (*p* > 0.05). However, no significant changes were observed in the granule cell layer in the cerebellum (Figure 2).

### 3.3. Effects of Pb Exposure and Ascorbic Acid Treatment on OPN and Brain-Derived Neurotrophic Factor (BDNF)Expression in the Developing Cerebellum

**Figure 3:**
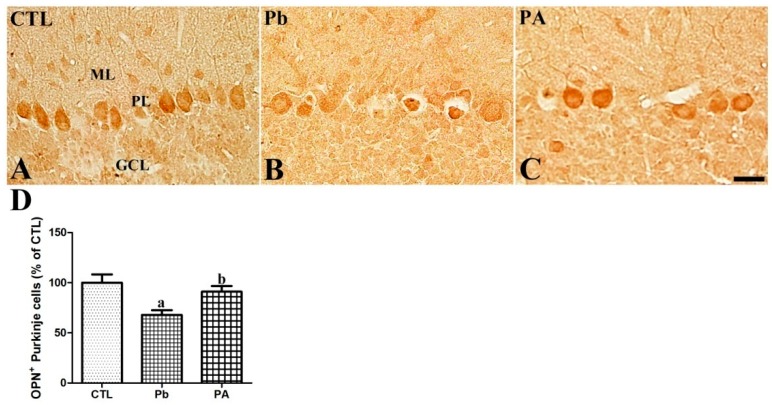
Immunohistochemistry for osteopontin (OPN) in the cerebellum (**A**–**C**) of offspring from CTL, Pb, and PA groups. GCL, granule cell layer; ML, molecular layer; PL, Purkinje cell layer. Bar = 25 μm. (**D**) The numbers of OPN-positive Purkinje cells in the cerebellar cortex are expressed as percentages of the value in the CTL group (*n* = 12 offspringper group; ^a^ *p* < 0.05, indicating a significant difference compared with the control group, ^b^ *p* < 0.05, indicating a significant difference compared with the Pb group). The bars indicate the means ± standard errors of the mean.

OPN was mainly detected in the Purkinje cell layer in the cerebellar cortex. Additionally, OPN was observed in some cells in the deep nucleus of the cerebellum in the offspring. After long-term exposure to Pb, the OPN-immunoreactive Purkinje cells were significantly reduced to 67.96% of that in the control group (*p* < 0.01). However, the co-administration of ascorbic acid attenuated the Pb-induced reduction in the OPN-immunoreactive Purkinje cells in the PA groupas91.01% of that in the control group (*p* < 0.05) and the mean number of these cells was similar to the control group (*p* > 0.05) (Figure 3). OPN was also detected in neuronal cells in the deep cerebellar nucleus, while OPN was not detected in the white matter of the cerebellum. The difference in the OPN expression, shape alteration, and degeneration in neuronal cells in the deep cerebellar nucleus was not observed among different groups (Figure S1). BDNF is animportant neurotrophic factor in brain development. To investigate the correlation between OPN and BDNF, Western blot analysis of OPN and BDNF in the whole cerebellum was conducted (Supplementary Method). The results showed a similar pattern of change; Pb-induced reduction of OPN (74.75% of that in the control group, *p* < 0.01) and BDNF (65.90% of that in the control group, *p* < 0.01) and ascorbic acid-mediated attenuation of the reduction (OPN, 94.64% of that in the control group; BDNF, 91.52% of that in the control group, *p* < 0.05) (Figure S2).

### 3.4. Effects of Pb Exposure and Ascorbic Acid Treatment on Olig2 Expression in the Developing Cerebellum

**Figure 4:**
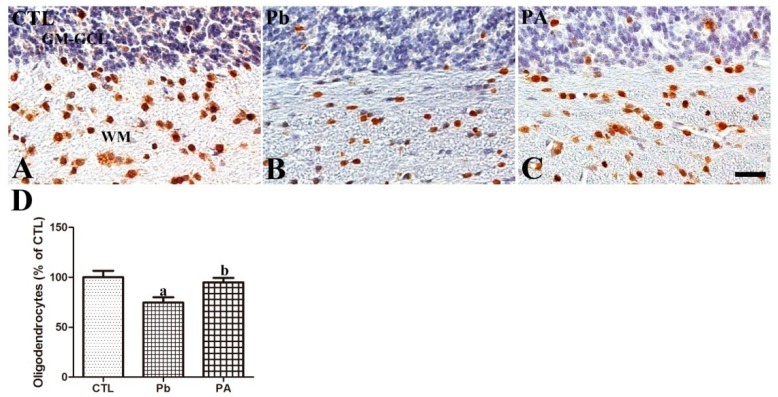
Immunohistochemistry for oligodendrocytes (brown) in the cerebellum (**A**–**C**) of offspring from CTL, Pb, and PA groups. Note that the numbers of Olig2-positive oligodendrocytes in the cerebellum are reduced in the Pb group, and ascorbic acid treatment ameliorated these reductions in the PA group. GM-GCL (blue), granule cell layer in gray matter; WM, white matter. Bar = 25 μm. (**D**) The numbers of OPN-positive Purkinje cells in the cerebellar cortex are expressed as percentages of the value in the CTL group (*n* = 12 offspring per group; ^a^ *p* < 0.05, indicating asignificant difference compared with the control group, ^b^ *p* < 0.05, indicating a significant difference compared with the Pb group). The bars indicate the means ± standard errors of the mean.

Olig2 was mainly detected in the white matter in the cerebellar cortex of offspring. Long-term exposure to Pb led to a significantly lower number of Olig2-immunoreactive oligodendrocytes than in the control group (74.72% of that in the control group, *p* < 0.05). Additionally, ascorbic acid treatment prevented the Pb-induced reduction in the number of Olig2-immunoreactive oligodendrocytes in the PA group (94.88% of that in the control group, *p* < 0.05). The mean number of Olig2-immunoreactive oligodendrocytes in the PA group was not significantly different from that in the control group (*p* > 0.05) (Figure 4).

### 3.5. Effect of Pb Exposure and Ascorbic Acid Treatment on MAG Expression in the Developing Cerebellum

**Figure 5:**
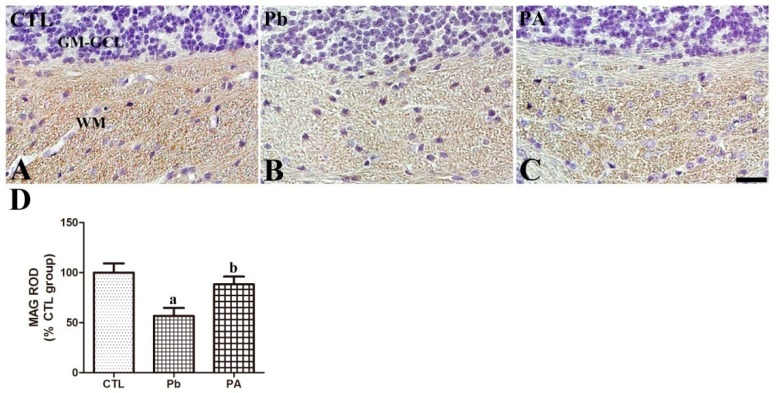
Immunohistochemistry for myelin-associated glycoprotein (MAG, brown) in the cerebellum (**A**–**C**) of offspring from CTL, Pb, and PA groups. Note that the MAG-immunoreactivity in the WM in the cerebellum are reduced in the Pb group, and ascorbic acid treatment ameliorated this reduction in the PA group. GM-GCL (blue), granule cell layer in gray matter. (**D**) The MAG-immunoreactivity in the WM in the cerebellum is expressed as percentages of the value in the CTL group (*n* = 12 offspring per group; ^a^ *p* < 0.05, indicating a significant difference compared with the control group, ^b^ *p* < 0.05, indicating a significant difference compared with the Pb group). The bars indicate the means ± standard errors of the mean.

MAG is transmembrane protein and is functionally classified as the protein component in the myelin sheath of nerve fibers [26]. In the cerebellum, MAG was commonly observed in the myelinated fibers of white matter tracts in all groups. The MAG immunoreactivity was reduced in the cerebellum of pups by prenatal and postnatal Pb exposure (57.66% of that in the control group, *p* < 0.01). However, ascorbic acid co-treatment with Pb ameliorated the Pb-induced reduction in the MAG immunoreactivity (88.35% of that in the control group, *p* < 0.05) to near the control level (*p* > 0.05) (Figure 5).

### 3.6. Effect of Pb Exposure and Ascorbic Acid Treatment on Locomotive Function (Bar Holding Test and Wire Mesh Ascending Test)

**Figure 6:**
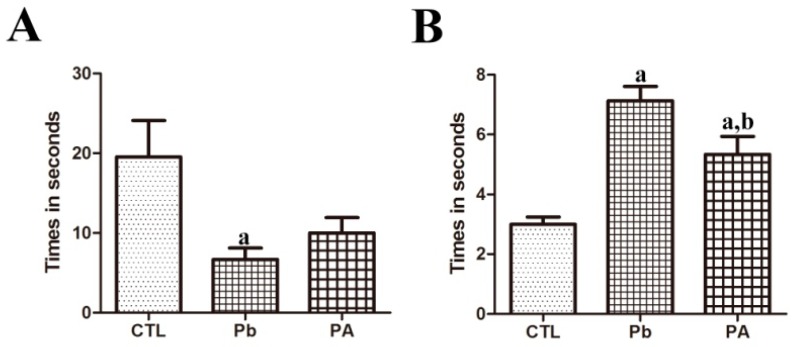
Effect of Pb exposure and ascorbic acid treatment in the bar holding test (**A**) and wire mesh ascending test (**B**) among offspring from CTL, Pb, and PA groups. (**A**) The times during which the animal stayed on the bar in the bar holding test. (**B**) The amount of time the animal spent to reach the top of the wire mesh in the wire mesh ascending test (*n* = 12 offspring per group; ^a^ *p* < 0.05, indicating a significant difference compared with the control group, ^b^ *p* < 0.05, indicating a significant difference compared with the Pb group).

The time spent grasping the bar was significantly reduced in pups of Pb-exposed dams than in those of the control group (control 19.13s; Pb 6.70 s) (*p* < 0.05), while ascorbic acid treatment slightly increased the time spent on the bar in the PA group (PA 10.00 s) (*p* > 0.05). The time of achievement of ascending the wire mesh was also delayed in the Pb-exposed offspring (control 3.10s; Pb 7.13 s) (*p* < 0.01) while ascorbic acid shortened the time of achievement with statistical significance in the PA group (PA 5.33 s) (*p* < 0.05). However, the time of achievement was still longer than the control group (*p* < 0.01) (Figure 6).

## 4. Discussion

The brain is susceptible to exogenous toxicants during the critical time window of development between the fetal period and the early postnatal period [27]. Specifically, Pb is highly toxic to the developing brain due to its ability to cross the blood–brain barrier [28]. OPN has been shown to be implicated during apoptosis, inflammation, degeneration, and cancer [2,4,9,29,30,31]. The pathological induction of OPN was observed among glial cells. However, the physiological expression of OPN in neurons has not been thoroughly studied [32,33]. The involvement of OPN in brain development was reported in a recent study by the Lönnerdal group [15]. Therefore, in the present study, we focused on the OPN expression in the developing cerebellum following Pb exposure.

We confirmed that long-term Pb treatment negatively affects normal development by significantly reducing cerebellar weight. Combined treatment of ascorbic acid with Pb attenuated the Pb-induced reduction in the weight of the cerebellum. The blood Pb level also showed a similar pattern of change. There was an increase in the blood Pb level and ascorbic acid administration attenuated the increase of Pb levels. The present findings are consistent with results of previous studies, in that ascorbic acid administration is conversely related to the blood Pb level after gestational Pb exposure [17,20]. However, the reduction of cerebellar weight was insignificant by gestational Pb exposure [17]. Exposure to Pb during the prenatal and postnatal periods, in the present study, was more detrimental than Pb exposure during the gestational period since it significantly affected these physiological parameters. While ascorbic acid treatment effectively reduced the negative effects of Pb on these parameters, it was unable to restore the Pb level to the control level.

Nissl staining revealed that gestational and lactational Pb exposure prominently impaired the development of the cerebellum. In the cerebellum, the number of intact Purkinje cells was significantly reduced by Pb exposure. Additionally, degenerating pyknotic cells and vacuolation in the Purkinje cell layer were easily detected in the Pb group. Similarly, previous studies reported that Pb exposure during cerebellar development significantly decreased the number of Purkinje cells and ascorbic acid treatment mitigated Pb-induced impairments in the PA group [17,20,34,35,36]. Our results are consistently similar with previous studies, which observed that ascorbic acid prevents the harmful effects of other heavy metals including mercury and cadmium on brain development [37,38].

We further evaluated the effect of Pb and ascorbic acid on OPN expression during cerebellar development. OPN was widely expressed in the developing brain in areas including the cerebellum, cortex, hippocampus, and pontine nucleus. Purkinje cells in the cortex and neurons in the deep nucleus in the developing cerebellum were OPN-immunoreactive cells. To investigate the association treatment of Pb and ascorbic acid with OPN expression during the developmental period, we compared cerebellar OPN expression among different experimental groups. The OPN immunoreactive Purkinje cells and OPN protein expression in the cerebellum were significantly reduced by long-term Pb exposure and ascorbic acid treatment attenuated this Pb-induced reduction. The morphology of OPN immunoreactive cells in the deep nucleus of the cerebellum was not different among the experimental groups. Additionally, the OPN immunoreactive cells in the pontine nucleus did not differ among the different experimental groups. Therefore, reduced cerebellar OPN protein is correlated with the reduction of OPN-immunoreactive Purkinje cells. The results presented here suggest that these Pb-induced developmental impairments and the ascorbic acid-mediated attenuation of cerebellar developmental impairments are associated with the change in OPN expression. Ascorbic acid-mediated increase in OPN in the cerebellum are linked with the positive effects of OPN in other studies which reported OPN-induced promotion of survival, proliferation, migration of neural stem cells and proliferation, and differentiation of glial cells into oligodendrocytes [15,39]. Additionally, OPN is required for normal retinal development, and OPN deficiency induces premature aging effects in the retina [40].

In a preliminary study on the neurotoxicity of Pb, Toews et al. [41] reported that myelin deficits in the developing rat brain were exacerbated in a Pb dose-dependent manner. Our previous study also demonstrated that gestational Pb exposure impaired the normal development of myelin sheaths by reducing the myelin basic protein (MBP) in the cerebellum [20,22]. Due to the role of OPN in neuronal axon myelination [15], we conducted further experiments to clearly demonstrate the effect of Pb on the myelination of neurons in the developing brain. We first investigated the changes in oligodendrocytes which are important for neuronal axon development and subsequent synapse formation. In the cerebellum, the number of Olig2-immunoreactive oligodendrocytes is reduced by Pb, and ascorbic acid co-treatment prevented this reduction. MAG is one of the main protein components of the myelin sheath and is required for the formation and stabilization of the myelin sheath [26]. Similar to MBP, MAG in the cerebellum was also affected by Pb exposure. Ultrastructurally, MAG is observed in oligodendrocyte processes at the axoglial junctions of the myelin sheath [26]. MAG immunochemistry revealed that Pb exposure-induced impairments in myelinated fibers in the developing cerebellum were attenuated by ascorbic acid co-administration. Along with the Pb-induced reduction of myelin-sheath forming oligodendrocytes and MAG, OPN may be directly associated with myelination process in the developing cerebellum. Although, the direct role of OPN in the axonal myelination was not demonstrated in the present study, Lönnerdal group’s recent study supports a correlation between OPN and axonal myelination by observing that milk OPN increased oligodendrocytes in the brain of OPN knockout mice while OPN-deprived milk reduced oligodendrocytes in the brain of OPN wild-type mice [15]. Selvaraju el al. also supports OPN’s role by reporting that OPN is upregulated during cuprizone-induced demyelination and remyelinating process, and that OPN treatment to cortical cultures increased the synthesis of MBP and formation of myelin sheath [42]. Besides the well-known pro-inflammatory role of OPN in adults, supplemented OPN to human infants has been shown to reduce pro-inflammatory cytokines, compared to the standard formula-fed infants [43]. Full-length OPN, the main type in milk, is different from forms of OPN in a pathological situation [44]. These recent studies, including our own, warrant the need for further studies to more thoroughly investigate OPN’s role in development and the possibility of OPN as biomarker molecule in the brain mal-development. Secondarily, Pb-induced myelin deficits may be linked with the change of OPN-immunoreactive Purkinje cells and the subsequent reduction of axonal fibers extended from soma of the Purkinje cells to the white matter in the cerebellum. Additionally, myelin sheath-associated structural changes have also been shown to be an important cause of multiple sclerosis, and these changes lead to functional deterioration [45]. Moreover, the down-regulation of MAG in the brain is associated with schizophrenia [46]. In Long Evans Shaker rats, the demyelination of Purkinje cells caused a reduction in gamma aminobutyric acid (GABA) released from Purkinje terminals, which resulted in the hyper-excitability of neurons in the deep cerebellar nucleus [47]. In the developing cortex of suckling rats, postnatal Pb consumption via diet also caused hypomyelination of neurons [48]. Similar to our results, ascorbic acid treatment promoted myelination in animal models and in cell cultures [20,49,50]. Based on these results, the present changes in the numbers of neurons and oligodendrocytes in the cerebellum are linked with differences in the MAG immunoreactive myelinated fibers in the Pb and PA groups.

As a mechanism of OPN expression related change in the developmental cerebellum, we focused on the BDNF for its role in processes including neurogenesis, synaptogenesis, axogenesis, and myelination [51,52]. Physiologically, OPN promotes the expression of pro-survival genes via nuclear factor kappa-b (NF-_K_B) [53]. BDNF is the target gene of NF-_K_B. Pb-induced reduction of BDNF was observed in our present and previous studies, and ascorbic acid was shown to increase the expression of BDNF [20,24].Along with the positive effect of ascorbic acid on BDNF expression, ascorbic acid is vital for neuronal differentiation and development, and myelin formation [54]. As vitamin B has a similar effect, such as the promotion of myelination and reduction of oxidative stress, co-administration of ascorbic acid with vitamin B as a multi-vitamin will help to protect the developing brain from Pb-induced neurotoxicity [55]. Additionally, the fact that remyelination of the nervous system is the goal of both the researcher and clinician [56], the mechanism of direct or indirect involvement of OPN in myelination will provide critical clues for development, regeneration, and therapy to demyelinating diseases.

Furthermore, we evaluated the locomotive function of the cerebellum via behavioral tests such as the wire mesh ascending test and bar holding test. The Pb-induced impairments in the developing cerebellum resulted in functional deterioration in the test. Pups of Pb-exposed dams demonstrated a longer latency to reach the top during the ascending wire mesh test. They were also unable to remain longer on the bar in the bar holding test compared to the control group. However, ascorbic acid treatment attenuated the Pb-induced functional impairment in these pups in the two behavioral tests. These improvements in the Pb-induced functional impairment may be due to ascorbic acid-mediated structural protection from Pb-induced alterations during the critical developmental period.

## 5. Conclusions

Overall, the present study demonstrated that Pb exposure is toxic to the developing cerebellum. It causes neuronal damage and reduces OPN expression in rat offspring. Ascorbic acid treatment reduced neuronal impairment and prevented Pb-induced changes. These results highlight the potential for Pb-induced developmental neurotoxicity and the protective effect of ascorbic acid co-administration in the cerebellum of the pups from mothers at high risk for Pb exposure during pregnancy and lactation.

## Supplementary Materials

The following are available online at https://www.mdpi.com/1660-4601/16/6/983/s1, Figure S1: Immunohistochemistry for osteopontin in the deep cerebellar nucleus and pontine nucleus, Figure S2: Western blot analysis of OPN and brain-derived neurotrophic factor (BDNF) in the cerebellum of offspring.

## References

[1] Denhardt D.T., Guo X. (1993). Osteopontin: A protein with diverse functions. FASEB J..

[2] Wang K.X., Denhardt D.T. (2008). Osteopontin: Role in immune regulation and stress responses. Cytokine Growth Factor Rev..

[3] Holm E., Gleberzon J.S., Liao Y., Sørensen E.S., Beier F., Hunter G.K., Goldberg H.A. (2014). Osteopontin mediates mineralization and not osteogenic cell development in vitro. Biochem. J..

[4] Chambers A.F., Behrend E.I., Wilson S.M., Denhardt D.T. (1992). Induction of expression of osteopontin (OPN; secreted phosphoprotein) in metastatic, ras-transformed NIH3T3 cells. Anticancer Res..

[5] Murry C.E., Giachelli C.M., Schwartz S.M., Vracko R. (1994). Macrophages express osteopontin during repair of myocardial necrosis. Am. J. Pathol..

[6] Goncalves R.F., Chapman D.A., Bertolla R.P. (2008). Pretreatment of cattle semen or oocyte with purified milk osteopontin affects in vitro fertilization and embryo development. Anim. Reprod. Sci..

[7] Liu Y., Xu H., Zhong W., Shen Q., Zhuang T., Huang K. (2015). Organic selenium alleviated the formation of ethylene glycol-induced calcium oxalate renal calculi by improving osteopontin expression and antioxidant capability in dogs. Biol. Trace Elem. Res..

[8] Moon C., Shin T. (2004). Increased expression of osteopontin in the spinal cords of Lewis rats with experimental autoimmune neuritis. J. Vet. Sci..

[9] Pio G.M., Xia Y., Piaseczny M.M., Chu J.E., Allan A.L. (2017). Soluble bone-derived osteopontin promotes migration and stem-like behavior of breast cancer cells. PLoS ONE.

[10] Sodek J., Ganss B., McKee M.D. (2000). Osteopontin. Crit. Rev. Oral. Biol. Med..

[11] Kim M.D., Cho H.J., Shin T. (2004). Expression of osteopontin and its ligand, CD44, in the spinal cords of Lewis rats with experimental autoimmune encephalomyelitis. J. Neuroimmunol..

[12] Niino M., Kikuchi S. (2011). Osteopontin and multiple sclerosis: An update. Clin. Exp. Neuroimmunol..

[13] Comi C., Carecchio M., Chiocchetti A., Nicola S., Galimberti D., Fenoglio C., Cappellano G., Monaco F., Scarpini E., Dianzani U. (2010). Osteopontin is increased in the cerebrospinal fluid of patients with Alzheimer’s disease and its levels correlate with cognitive decline. J. Alzheimers Dis..

[14] Mattson N., Rüetschi U., Pijnenburg Y.A., Blankenstein M.A., Podust V.N., Li S., Fagerberg I., Rosengren L., Blennow K., Zetterberg H. (2008). Novel cerebrospinal fluid biomarkers of axonal degeneration in frontotemporal dementia. Mol. Med. Rep..

[15] Jiang R., Prell C., Lönnerdal B. (2018). Milk osteopontin promotes brain development by up-regulating osteopontin in the brain in early life. FASEB J..

[16] Chang B.J., Jang B.J., Son T.G., Cho I.H., Quan F.S., Choe N.H., Nahm S.S., Lee J.H. (2012). Ascorbic acid ameliorates oxidative damage induced by maternal low-level lead exposure in the hippocampus of rat pups during gestation and lactation. Food Chem. Toxicol..

[17] Nam S.M., Chang B.J., Kim J.H., Nahm S.S., Lee J.H. (2018). Ascorbic acid ameliorates lead-induced apoptosis in the cerebellar cortex of developing rats. Brain Res..

[18] World Health Organization (2010). Childhood Lead Poisoning. http://www.who.int/ceh/publications/childhoodpoisoning/en/.

[19] Lee M.Y., Choi J.S., Lim S.W., Cha J.H., Chun M.H., Chung J.W. (2001). Expression of osteopontin mRNA in developing rat brainstem and cerebellum. Cell Tissue Res..

[20] Nam S.M., Cho I.S., Seo J.S., Go T.H., Kim J.H., Nahm S.S., Chang B.J., Lee J.H. (2019). Ascorbic acid attenuates lead-induced alterations in the synapses in the developing rat cerebellum. Biol. Trace Elem. Res..

[21] Lee J.Y., Chang M.Y., Park C.H., Kim H.Y., Kim J.H., Son H., Lee Y.S., Lee S.H. (2003). Ascorbate-induced differentiation of embryonic cortical precursors into neurons and astrocytes. J. Neurosci. Res..

[22] Nam S.M., Seo J.S., Go T.H., Nahm S.S., Chang B.J. (2018). Ascorbic acid supplementation prevents the detrimental effects of prenatal and postnatal lead exposure on the Purkinje cell and related proteins in the cerebellum of developing rats. Biol. Trace Elem. Res..

[23] Perez-Polo J., Rea H.C., Infante S.K. (2015). Locomotor coordination assay in rats. Bio-protocol.

[24] Nam S.M., Seo M., Seo J.-S., Rhim H., Nahm S.-S., Cho I.-H., Chang B.-J., Kim H.-J., Choi S.-H., Nah S.-Y. (2019). Ascorbic acid mitigates D-galactose-induced brain aging by increasing hippocampal neurogenesis and improving memory function. Nutrients.

[25] Volk B. (1984). Cerebellar histogenesis and synaptic maturation following pre-and postnatal alcohol administration. Acta Neuropathol..

[26] Quarles R.H. (2007). Myelin-associated glycoprotein (MAG): Past, present and beyond. J. Neurochem..

[27] Lanphear B.P. (2015). The impact of toxins on the developing brain. Annu. Rev. Public Health.

[28] Wang Q., Luo W., Zheng W., Liu Y., Xu H., Zheng G., Dai Z., Zhang W., Chen Y., Chen J. (2007). Iron supplement prevents lead-induced disruption of the blood-brain barrier during rat development. Toxicol. Appl. Pharmacol..

[29] Kim H.L., Chang B.J., Nam S.M., Nahm S.S., Lee J.H. (2017). Increased osteopontin expression and mitochondrial swelling in 3-nitropropionic acid-injured rat brains. Rom. J. Morphol. Embryol..

[30] Maetzler W., Berg D., Schalamberidze N., Melms A., Schott K., Mueller J.C., Liaw L., Gasser T., Nitsch C. (2007). Osteopontin is elevated in Parkinson’s disease and its absence leads to reduced neurodegeneration in the MPTP model. Neurobiol. Dis..

[31] Scatena M., Liaw L., Giachelli C.M. (2007). Osteopontin: A multifunctional molecule regulating chronic inflammation and vascular disease. Arterioscler. Thromb. Vasc. Biol..

[32] Choi J.S., Park H.J., Cha J.H., Chung J.W., Chun M.H., Lee M.Y. (2003). Induction and temporal changes of osteopontin mRNA and protein in the brain following systemic lipopolysaccharide injection. J. Neuroimmunol..

[33] Choi J.S., Kim H.Y., Cha J.H., Choi J.Y., Lee M.Y. (2007). Transient microglial and prolonged astroglial upregulation of osteopontin following transient forebrain ischemia in rats. Brain Res..

[34] Eltony S.A., Othman M.A., Mohamed A.A. (2010). Histological study on the effect of low level perinatal lead exposure on the cerebellar cortex of adult male albino rat. Egypt J. Histol..

[35] Mustafa H.N., Hussein A.M. (2016). Does allicin combined with vitamin B-complex have superior potentials than alpha-tocopherol alone in ameliorating lead acetate-induced Purkinje cell alterations in rats? An immunohistochemical and ultrastructural study. Folia Morphol..

[36] Saleh H.A., Abdel El-Aziz G.S., Mustafa H.N., Saleh A.H.A., Mal A.O., Deifalla A.H.S., Abo Rass M. (2018). Protective effect of garlic extract against maternal and fetal cerebellar damage induced by lead administration during pregnancy in rats. Folia Morphol. (Warsz).

[37] El-Sokkary G.H., Awadalla E.A. (2011). The protective role of vitamin C against cerebral and pulmonary damage induced by cadmium chloride in male adult albino rat. Open Neuroendocrinol. J..

[38] Ibegbu A.O., Abdulrazaq A.A., Micheal A., Daniel B., Sadeeq A.A., Peter A., Hamman W.O., Umana U.E., Musa S.A. (2014). Histomorphological effect of ascorbic acid on mercury chloride-induced changes on the cerebellum of adult wistar rats. J. Morphol. Sci..

[39] Rabenstein M., Hucklenbroich J., Willuweit A., Ladwig A., Fink G.R., Schroeter M., Langen K.J., Rueger M.A. (2015). Osteopontin mediates survival, proliferation and migration of neural stem cells through the chemokine receptor CXCR4. Stem Cell Res. Ther..

[40] Ruzafa N., Pereiro X., Aspichueta P., Araiz J., Vecino E. (2018). The retina of osteopontin deficient mice in aging. Mol. Neurobiol..

[41] Toews A.D., Krigman M.R., Thomas D.J., Morell P. (1980). Effect of inorganic lead exposure on myelination in the rat. Neurochem. Res..

[42] Selvaraju R., Bernasconi L., Losberger C., Graber P., Kadi L., Avellana-Adalid V., Picard-Riera N., Baron Van Evercooren A., Cirillo R., Kosco-Vilbois M. (2004). Osteopontin is upregulated during in vivo demyelination and remyelination and enhances myelin formation in vitro. Mol. Cell. Neurosci..

[43] Demmelmair H., Prell C., Timby N., Lönnerdal B. (2017). Benefits of lactoferrin, osteopontin and milk fat globule membranes for infants. Nutrients.

[44] Christensen B., Sorensen E.S. (2016). Structure, function and nutritional potential of milk osteopontin. Int. Dairy J..

[45] Smith K.J., McDonald W.I. (1999). The pathophysiology of multiple sclerosis: The mechanisms underlying the production of symptoms and the natural history of the disease. Philos. Trans. R. Soc. Lond. B. Biol. Sci..

[46] Felsky D., Voineskos A.N., Lerch J.P., Nazeri A., Shaikh S.A., Rajji T.K., Mulsant B.H., Kennedy J.L. (2012). Myelin-associated glycoprotein gene and brain morphometry in schizophrenia. Front. Psychiatry.

[47] Barron T., Saifetiarova J., Bhat M.A., Kim J.H. (2018). Myelination of Purkinje axons is critical for resilient synaptic transmission in the deep cerebellar nucleus. Sci. Rep..

[48] Krigman M.R., Druse M.J., Traylor T.D., Wilson M.H., Newell L.R., Hogan E.L. (1974). Lead encephalopathy in the developing rat: Effect upon myelination. J. Neuropathol. Exp. Neurol..

[49] Eldridge C.F., Bunge M.B., Bunge R.P., Wood P.M. (1987). Differentiation of axon-related Schwann cells in vitro. I. Ascorbic acid regulates basal lamina assembly and myelin formation. J. Cell Biol..

[50] Kim J.Y., Choi C.S., Hong S.K. (2014). Coculture of Schwann cells and neuronal cells for myelination in rat. Rapid Commun. Photosci..

[51] Kelamangalath L., Smith G.M. (2013). Neurotrophin treatment to promote regeneration after traumatic CNS injury. Front. Biol..

[52] Fletcher J.L., Murray S.S., Xiao J. (2018). Brain-derived neurotrophic factor in central nervous system myelination: A new mechanism to promote myelin plasticity and repair. Int. J. Mol. Sci..

[53] Zhao H., Chen Q., Alam A., Cui J., Suen K.C., Soo A.P., Eguchi S., Gu J., Ma D. (2018). The role of osteopontin in the progression of solid organ tumour. Cell Death Dis..

[54] Travica N., Ried K., Sali A., Scholey A., Hudson I., Pipingas A. (2017). Vitamin C status and cognitive function: A systematic review. Nutrients.

[55] Ford T.C., Downey L.A., Simpson T., McPhee G., Oliver C., Stough C. (2018). The effect of a high-dose vitamin B multivitamin supplement on the relationship between brain metabolism and blood biomarkers of oxidative stress: A randomized control trial. Nutrients.

[56] Franklin R.J., Ffrench-Constant C. (2008). Remyelination in the CNS: From biology to therapy. Nat. Rev. Neurosci..

