# Supplementary material for: Effects of Ascorbic Acid on Osteopontin Expression and Axonal Myelination in the Developing Cerebellum of Lead-Exposed Rat Pups
Source: Int J Environ Res Public Health. 2019 Mar 19;16(6):983. doi: 10.3390/ijerph16060983 (PMC6466450; doi:10.3390/ijerph16060983)
Supplement: Supplementary file 1 [file ijerph-16-00983-s001.pdf]

International Journal of
Environmental Research and Public Health

Supplementary Data: Effects of Ascorbic Acid on Osteopontin Expression and Axonal Myelination in the Developing Cerebellum of Lead-Exposed Rat Pups

Sung Min Nam, Jin Seok Seo, Sang-Soep Nahm and Byung-Joon Chang *

Department of Anatomy, College of Veterinary Medicine, Konkuk University, Seoul 05030, Korea; skavet@konkuk.ac.kr (S.M.N.); phoenix_1st@naver.com (J.S.S.); ssnahm@konkuk.ac.kr (S.-S.N.)

* Correspondence: bjchang@konkuk.ac.kr; Tel.: +82-2-450-3711; Fax: +82-2-450-3037

Received: 30 January 2019; Accepted: 14 March 2019; Published: 19 March 2019

Supplementary Methods

Immunoblotting Assay

To confirm the effects of lead (Pb) and ascorbic acid on the expression level of osteopontin (OPN) and brain-derived neurotrophic factor (BDNF) in the cerebellum, 12 rat offspring in each group were sacrificed by decapitation after urethane (2 g/kg) anesthesia. Brains were quickly removed, and cerebella were then dissected with a surgical blade, weighed, and stored at -80°C for analysis. For immunoblotting, cerebellar tissues were homogenized in 50 mM PBS (pH 7.4) containing 0.1 mM ethyleneglycol bis (2-aminoethyl ether)-N,N,N′,N′ tetraacetic acid (EGTA) (pH 8.0), 0.2% nonidet P-40, 10 mM ethylendiamine tetraacetic acid (EDTA) (pH 8.0), 15 mM sodium pyrophosphate, 100 mM β-glycerophosphate, 50 mM NaF, 150 mM NaCl, 2 mM sodium orthovanadate, 1 mM phenylmethylsulfonyl fluoride (PMSF) and 1 mM dithiothreitol (DTT). After centrifugation, protein concentration in the supernatants was determined using a Micro Bicinchoninic acid (BCA) protein assay kit with bovine serum albumin as the standard (Pierce Chemical, Rockford, IL, USA). Aliquots containing 80 μg proteins were denatured in loading buffer containing 150 mM Tris (pH 6.8), 3 mM DTT, 6% SDS, 0.3% bromophenol blue, and 30% glycerol. The aliquots were then loaded onto 10% polyacrylamide gel. After electrophoresis, the gels were transferred to nitrocellulose transfer membranes (Pall Corp., East Hills, NY, USA). To reduce background staining, the membranes were incubated with 5% skim milk in Tris-buffered saline (TBS, pH 7.4) containing 0.1% Tween 20 for 1 h, followed by incubation with primary antibody against OPN (goat, 1:1,000; R&D systems, Minneapolis, MN, USA), or BDNF (rabbit, 1:2,000, Novus, Littleton, CO, USA) overnight at 4°C. The blots were washed three times in TBS containing 0.1% Tween-20 and then incubated with horseradish peroxidase-conjugated anti-goat or anti-rabbit IgG (1:2000). Bands were visualized using SuperSignal® West Pico Chemiluminescent Substrate (Thermo Fisher Scientific, Rockford, IL, USA). The blot was densitometrically scanned for the quantification of relative optical density (ROD) of each band using NIH Image 1.59 software. The data were normalized against β-actin.

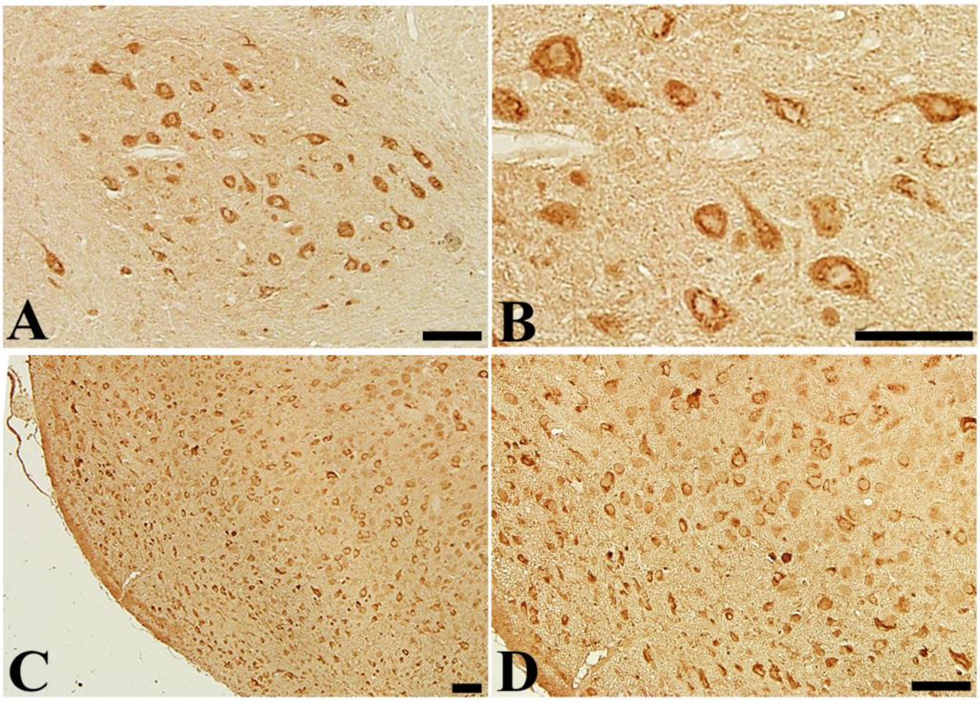

Figure S1. Immunohistochemistry for osteopontin (OPN) in the deep cerebellar nucleus and pontine nucleus (A, B, C, and D) of offspring from control (CTL) rats. Bar = 50 μm.

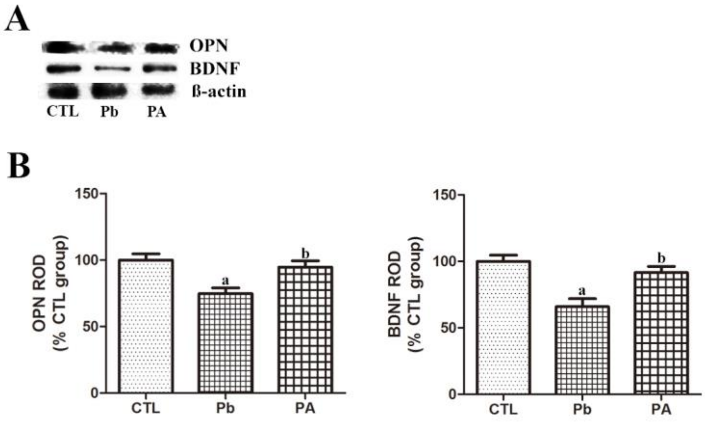

Figure S2. Western blot analysis of OPN and brain-derived neurotrophic factor (BDNF) in the cerebellum of offspring from CTL, lead (Pb), and Pb + ascorbic acid (PA) groups. Relative optical density (ROD) of OPN and BDNF immunoblot bands is defined as a percentage of the value of the control group (n = 12 per group; $^{a}p < 0.05$, indicating a significant difference compared with the control group, $^{b}p < 0.05$, indicating a significant difference compared with the Pb group). The bars indicate the means ± standard errors of the mean.
